# Solution Structure, Copper Binding and Backbone Dynamics of Recombinant Ber e 1–The Major Allergen from Brazil Nut

**DOI:** 10.1371/journal.pone.0046435

**Published:** 2012-10-04

**Authors:** Louise Rundqvist, Tobias Tengel, Janusz Zdunek, Erik Björn, Jürgen Schleucher, Marcos J. C. Alcocer, Göran Larsson

**Affiliations:** 1 Department of Medical Biochemistry and Biophysics, Umeå University, Umeå, Sweden; 2 Protein Constructor Developers Company, Umeå, Sweden; 3 Department of Chemistry, Umeå University, Umeå, Sweden; 4 Department of Nutritional Sciences, University of Nottingham, Loughborough, United Kingdom; George Washington University, United States of America

## Abstract

**Background:**

The 2S albumin Ber e 1 is the major allergen in Brazil nuts. Previous findings indicated that the protein alone does not cause an allergenic response in mice, but the addition of components from a Brazil nut lipid fraction were required. Structural details of Ber e 1 may contribute to the understanding of the allergenic properties of the protein and its potential interaction partners.

**Methodology/Principal Findings:**

The solution structure of recombinant Ber e 1 was solved using NMR spectroscopy and measurements of the protein back bone dynamics at a residue-specific level were extracted using ^15^N-spin relaxation. A hydrophobic cavity was identified in the structure of Ber e 1. Using the paramagnetic relaxation enhancement property of Cu^2+^ in conjunction with NMR, it was shown that Ber e 1 is able to specifically interact with the divalent copper ion and the binding site was modeled into the structure. The IgE binding region as well as the copper binding site show increased dynamics on both fast ps-ns timescale as well as slower µs-ms timescale.

**Conclusions/Significance:**

The overall fold of Ber e 1 is similar to other 2S albumins, but the hydrophobic cavity resembles that of a homologous non-specific lipid transfer protein. Ber e 1 is the first 2S albumin shown to interact with Cu^2+^ ions. This Cu^2+^ binding has minimal effect on the electrostatic potential on the surface of the protein, but the charge distribution within the hydrophobic cavity is significantly altered. As the hydrophobic cavity is likely to be involved in a putative lipid interaction the Cu^2+^ can in turn affect the interaction that is essential to provoke an allergenic response.

## Introduction

Despite the enormous diversity of the human diet, relatively few foods products are able to sensitize and elicit an allergic reaction. Today, around 400 food allergens have been identified, clustering to only 0.6% of all currently known protein families [1], [2], [3]. In plants, the prolamin superfamily contains the largest number of allergenic proteins [4], comprising proteins such as trypsin-alpha amylase inhibitors, lipid transfer proteins and 2S albumins. The 2S albumins in particular are water soluble proteins present in seeds of a wide range of species. They are characterized by their conserved structure of antiparallel bundles of four helices held together by four disulfide bonds in a distinctive right-handed superhelix fold and can, in some cases, be very rich in sulfur containing amino acids [5], [6].

For the assessment of food safety, it is important to characterize the molecular details that distinguish food allergens from non-allergens. The Brazil nut, *Bertholletia excelsa* (BN) 2S albumin, Ber e 1, is described as the major allergen in BN [7], and the protein has been linked to several anaphylactic reactions leading to fatalities [8]. Historically, Ber e 1 has often been used as an example of the unintentional consequences that can occur in genetically modified (GM) crops. Ber e 1 consists of roughly 25% sulfurous amino acids [9], [10] and for this reason its gene was cloned into soybean with the intention of boosting the sulfur content of this leguminous plant. The outcome attracted much media attention since BN-allergic patients showed positive reactions in a skin-prick test (SPT) to the transgenic soybean, but not to the unmodified soybean, making Ber e 1 the first allergen to be transferred from one plant to another [11].

Stored in the hypocotyls of the embryo, wild-type Ber e 1 is posttranslationally cleaved into a small and large subunit that is linked together by four disulfide bonds [10], [12]. In order to perform structural studies, a recombinant Ber e 1 protein was overexpressed in the methylotropic yeast *Pichia pastoris* [12]. The recombinant Ber e 1 (rBer e 1) was expressed as a single polypeptide chain, similar to the wt protein, and was shown to contain partial posttranslational processing instead of the total removal of the linker peptide between the small and large subunits as observed in the wt protein [10]. In addition the rBer e 1, when expressed in *Pichia pastoris*, becomes O-glycosylated. Nevertheless, rBer e 1 shares the same disulfide pattern, extreme high stability and fold as the wild-type protein [12].

Several traits such as high thermal stability, pH tolerance and resistance towards proteolytic degradation have been used to characterize food allergens [1], however, these characteristics are not unique to allergenic proteins. Within the 2S albumin family, major allergens as well as proteins not implicated in allergy can be found. These proteins are structurally similar, possess comparable biophysical characteristics but also possess great variation in amino acid sequence [10].

In addition to the intrinsic properties of allergenic proteins, extrinsic factors present in the food matrix may also participate in the development of an allergic response [13]. In fact, purified Ber e 1 requires the addition of a BN lipid fraction in order to induce an allergic reaction in mice [14]. Ber e 1, as we have previously shown by NMR, is able to directly interact with undefined components of the immunoactive lipid fraction [10]. This characteristic is not particular to Ber e 1 and has been observed for other 2S albumins. For example, Sin a 1, a 2S albumin from mustard seeds, shows affinity for several different phospholipids [15]. It has also been shown that purified allergenic proteins from peanut were not able to induce a significant immune activation while the whole food extract, including the allergens, was immunoactive [16].

While Ber e 1 is capable of hydrophobic interactions with natural lipids, less is known about its ability to make electrostatic interactions with metal ions. However, it has been reported that a water soluble protein fraction from BN has an unusually high concentration of divalent ions such as Cu^2+^, Fe^2+^, Mn^2+^ and Zn^2+^ [17].

Structural changes in proteins occurs on different timescales, ranging from fast backbone and side chain dynamics within a small conformational space, to slower large scale transitions between conformational states involving the protein backbone. Slower dynamics at the µs-ms timescale is frequently related to biological binding activity [18], [19], [20]. Dynamics on both the fast (ps-ns) and slow (µs-ms) timescales can be successfully accessed by NMR.

In this article, the solution structure of Ber e 1 is presented, which has been solved using NMR spectroscopy, along with measurement of the protein backbone dynamics at a residue-specific level. We also show that Ber e 1 is able to specifically interact with the divalent copper ion with a 1∶1 stoichiometry and high affinity. Using the paramagnetic relaxation enhancement property of Cu^2+^, the approximate copper binding site was modeled close to the bottom of a deep hydrophobic cavity within the structure of Ber e 1. The binding of the Cu^2+^ ion has surprisingly low effect on the electrostatic potential on the surface of the protein, but significantly alter the charge distribution within the hydrophobic cavity of Ber e 1.

## Results and Discussion

### Structure of Ber e 1

Ber e 1 has previously been identified as a helical protein using CD spectroscopy [12] as well as NMR spectroscopy [9]. wt Ber e 1 is encoded by several polymorphic genes and the final proteins are all posttranslationally processed [21]. Recombinant Ber e 1 contains a peptide sequence that is normally posttranslationally cleaved off in the wt protein [10], [12], [21] in the Ber e 1 structure presented here this corresponds to a loop consisting of residues 33–38 connecting helix 1b and 2. In the NMR structure ensemble these residues are in a random coil conformation, and have no NMR-detectable NOE distances to the core of the protein. Hence we conclude that the sequence has negligible influence on the structure.

**Figure 1:**
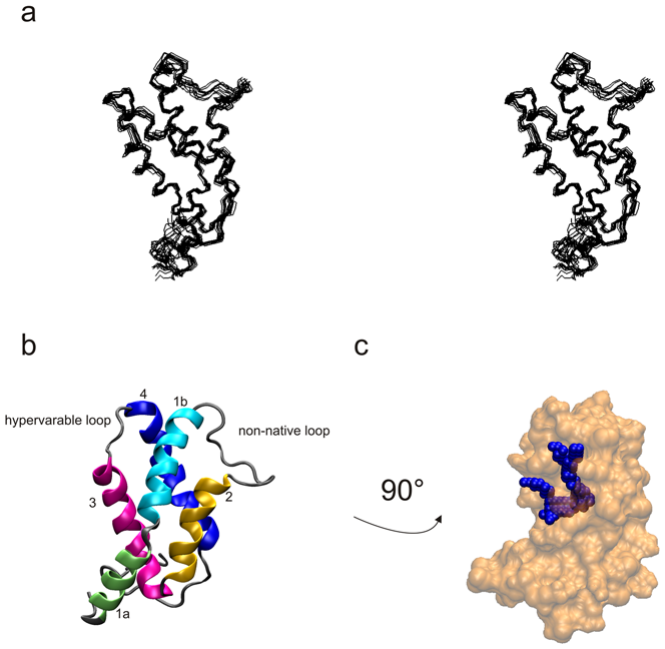
Solution structure of Ber e 1. a) Stereo view of the backbone of the 12 lowest energy structures after energy minimization. b) Cartoon representation of the Ber e 1 structure, with the different structure elements colored as follows: green = helix 1a, cyan = helix 1b, yellow = helix 2, pink = helix 3, blue = helix 4. c) Location of the hydrophobic cavity in Ber e 1. The structure has been rotated 90 degrees with respect to figure 1a and b.

The backbone trace of an NMR structure ensemble of the 12 lowest energy structures of rBer e 1 is presented in Figure 1a. Statistical details of the 12 final structures are summarized in Table 1. The structures show good geometry and are consistent with assigned NOEs, ^3^J_HN-Hα_ coupling constants and chemical shifts. The structure of rBer e 1 resembles that of other known 2S albumins such as Ric c 3 [22], Ara h 6 [23] Napin [24] pronapin [25], and SFA8 [26], which all fall within a backbone rmsd between 2.4–4.7 Å, adding evidence for the evolutionary conserved fold of 2S albumins. A cartoon representation of the average structure is shown in Figure 1b. The few known structures of 2S albumins have a common fold of a four helix, disulfide rich, right handed super helix [27], with a segment connecting helix 3 and 4 known as the hypervariable region, which is the immunodominant part of the protein [6], [28], [29], [30]. Atomic coordinates of the structure ensemble of Ber e 1 has been deposited to the RCSB protein data bank (http://www.pdb.org) under the accession code 2LVF.

**Table 1:** Structural statistics of the 12 lowest energy structures.

NMR derived constraints	Number of constraints
Total distances (NOE)	1038
Intraresidual distances (i = j)	606
Sequential distances (|i−j| = 1)	329
Short range distances (1<|i−j|≤4)	92
Long range distances (|i−j|>4)	11
Dihedral angle restraints	226
J-coupling constant restraints	80
Chemical shifts for CA/CB	166

Constraints violations	rmsd	Total number of violations
Bonds (Å^2^)	0.004	(0)
Angles (°)	0.595	(0)
NOE	0.068	(5) (# of viol. >0.5 Å)
Dihedral angle restraints	1.136	(8) (# of viol. >5°)
J-coupling constant restraints	1.012	(24) (# of viol. >1 Hz)
Chemical shifts for CA/CB	1.322/0.953	(62) (# of viol. >1 ppm)

Energies of final structures	Average (kcal/mol)	std. dev (kcal/mol)
bond	24.24	3.02
angle	176.97	8.49
impr	32.80	2.61
vdw	−545.04	10.83
rama	−1354.18	18.78
cdih	28.94	18.82
noe	148.36	16.41
jcoup	82.54	15.92
ccs CA/CB	105.09	1.97
total	−1405.37	66.38

RMSD comparison of backbone atoms	Average (Å)	std. dev (Å)
residues 5∶30 and 45∶105	0.695	0.112

Ber e 1 requires the addition of a lipid fraction in order to provoke an allergic response in mice [14], and the lipids in the immunoactive fraction consist of fatty acid chains with lengths of 16 and 18 carbons as determined by gas-chromatography (data not shown). NMR experiments have shown that the addition of lipids alters the ^1^H-^15^N HSQC spectrum, indicating a direct interaction between one or more components of the immunoactive fraction [10]. A hydrophobic binding site forming a deep cavity was modeled *in silico* [31] and was identified in all structures within the ensemble. The residues forming the cavity are MET17, CYS21, TYR24, CYS49, LEU53, LEU65, ARG66, MET68, MET69, MET72, MET88, ARG89, ALA91, GLU92, ILE94 and PRO95, which are mainly of hydrophobic character. The entrance of the cavity is located between helix 3 and 4, penetrating trough the core of the protein (Figure 1c), and is approximately 16 Å deep with a volume of 75–90 Å^3^, depending on the varied orientation of the amino acid side chains within the structure ensemble. Therefore, the cavity is both chemically suitable, as well as appropriate in size to accommodate a fatty acid chain from the immunoactive lipid fraction. It is interesting to note that the hydrophobic cavity of Ber e 1 is located analogously to the lipid binding site in the type 2 non-specific lipid transfer protein (ns-LTP) from wheat (PDB code: 1N89 [32]). In these proteins the fatty acid side chain of L-alpha-palmitoylphosphatidylglycerol (LPG) enters the ns-LTP between helix 3 and 4 and is of approximately the same depth as the hydrophobic cavity in Ber e 1.

**Figure 2:**
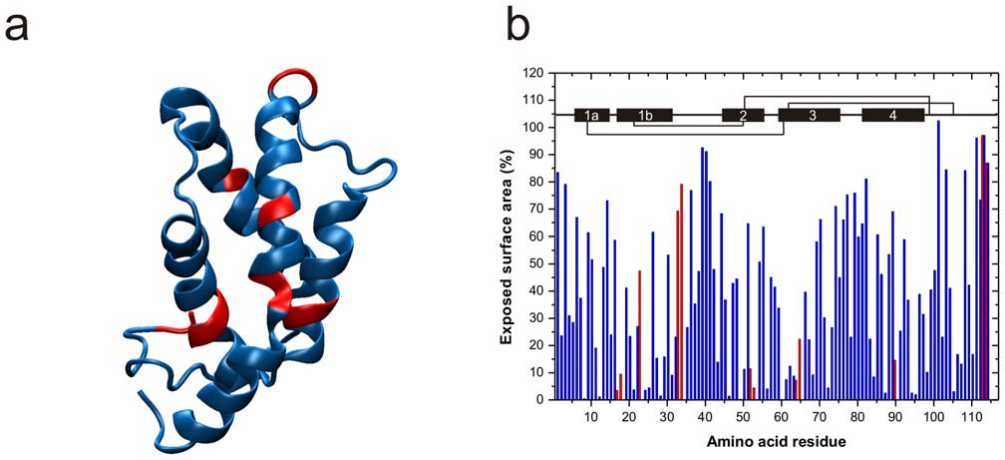
Theroretical pepsin cleavage sites and solvent exposed residues. a) Theoretical pepsin cleavage sites (red) mapped on the tertiary structure of Ber e 1. b) Exposed surface area of the N and H^N^ atoms in the backbone of Ber e 1. Residues able to undergo pepsin cleavage are highlighted in red. The secondary structure elements, as well as the cysteine linkage are indicated in the top of the figure. Most of the theoretical pepsin cleavage sites are buried within the α-helices. The only surface exposed pepsin cleavage sites are located in the non-native loop and the C-terminal, and cleavage at these positions would not disrupt the integrity of the structure.

Food allergens are frequently described as very stable proteins, surviving processes of heating and exposure to gastric fluid that would normally cause unfolding of the native state or degradation. The physiological stability of Ber e 1 was particularly demonstrated in a rather extreme case, when the semen of a man who orally ingested Brazil nuts caused an allergic reaction in a BN allergic patient [33]. The high chemical and thermal stability of recombinant and wild-type Ber e 1 has also been demonstrated in vitro [34] suggesting that Ber e 1 is the only BN protein able to reach the gut mucosa as an intact protein. In the gut proteins are denatured by the low pH, and subsequently digested to peptide fragments by Pepsin. The theoretical pepsin cleavage sites of Ber e 1 were mapped on the tertiary structure (Figure 2a). A majority of cleavage sites are protected in the interior of the protein, as shown in Figure 2b, and in the cases where pepsin may reach a cleavage site, the four disulfide bonds are likely to maintain the overall fold of the protein [35]. These protected cleavage sites together with the compact fold held together with the four disulfide bridges offer an explanation to the high resistance to Ber e 1 digestion in the rough environment of the gastric fluid.

### Comparison with other proteins

The three-dimensional structure of Ber e 1 was compared to other known protein structures using the DALI server [36], which compares protein three-dimensional structures without taking sequence homology as a prerequisite. As expected from the SCOP database [27], the structure of Ber e 1 has a fold similar to other 2 s albumins, ns-LTPs and amylase inhibitors. However, a vast majority of proteins found to be structurally similar to Ber e 1 are non-homologous and have not been identified as allergens. For example, the 2S albumin fold can appear as a single domain, as in the case of mabinlin, an artificial sweetener, or as a domain in a larger protein such as the C-terminal domain of *Thermosynechococcus elongatus* circadian clock protein KaiA. Aside from the similarity in their tertiary structure, many of these proteins have the common trait of extreme temperature tolerance.

It has previously been suggested that there is no common tertiary structure among allergenic proteins [37], which is further supported by the many structurally similar non-allergens identified in the DALI search. Despite this, only 0.6% of protein families are known to cause allergic reactions [2]. This implies that the allergenic properties of a protein are not determined by a single attribute such as backbone fold or linear epitopes, but much more complex intrinsic and extrinsic factors that are recognized by the immune system of susceptible individuals. In the case of food allergens, the protein stability is most likely one factor, but certainly not sufficient alone, because many 2S albumins are stable yet non-allergenic. Clearly, other factors must contribute to render a protein allergenic. Good candidates for such factors are dynamical, electrostatic and hydrophobic properties; their identification would improve our understanding of allergic responses.

### Backbone Dynamics

Protein interaction is a dynamical process where the backbone adopts different conformational states in order to accommodate a ligand. These conformational states may be discrete and difficult to detect by traditional methods of structure determination. Biological binding activities normally occur at a timescale of µs to ms, which can be accessed by NMR spin relaxation measurements. Faster dynamics at the ps-ns timescale can also be readily measured by NMR and can be used to explain the rotational freedom of the N-H bond vector, which is useful to determine the overall rigidity of the backbone at a residue-specific basis.

To our knowledge, no reports have been made on the backbone dynamics of 2 s albumins. It is therefore interesting to determine the backbone dynamics of Ber e 1 in order to elucidate what kind of dynamical processes occur in a 2 s albumin. In all known 2S albumin structures the immunodominant hypervariable loop region seems to be surprisingly well defined despite its solvent exposed nature [22], [23], [24], [25], [26]. Thus the characterization of the dynamics in such regions can provide details of the recognition of IgE antibodies.

**Figure 3:**
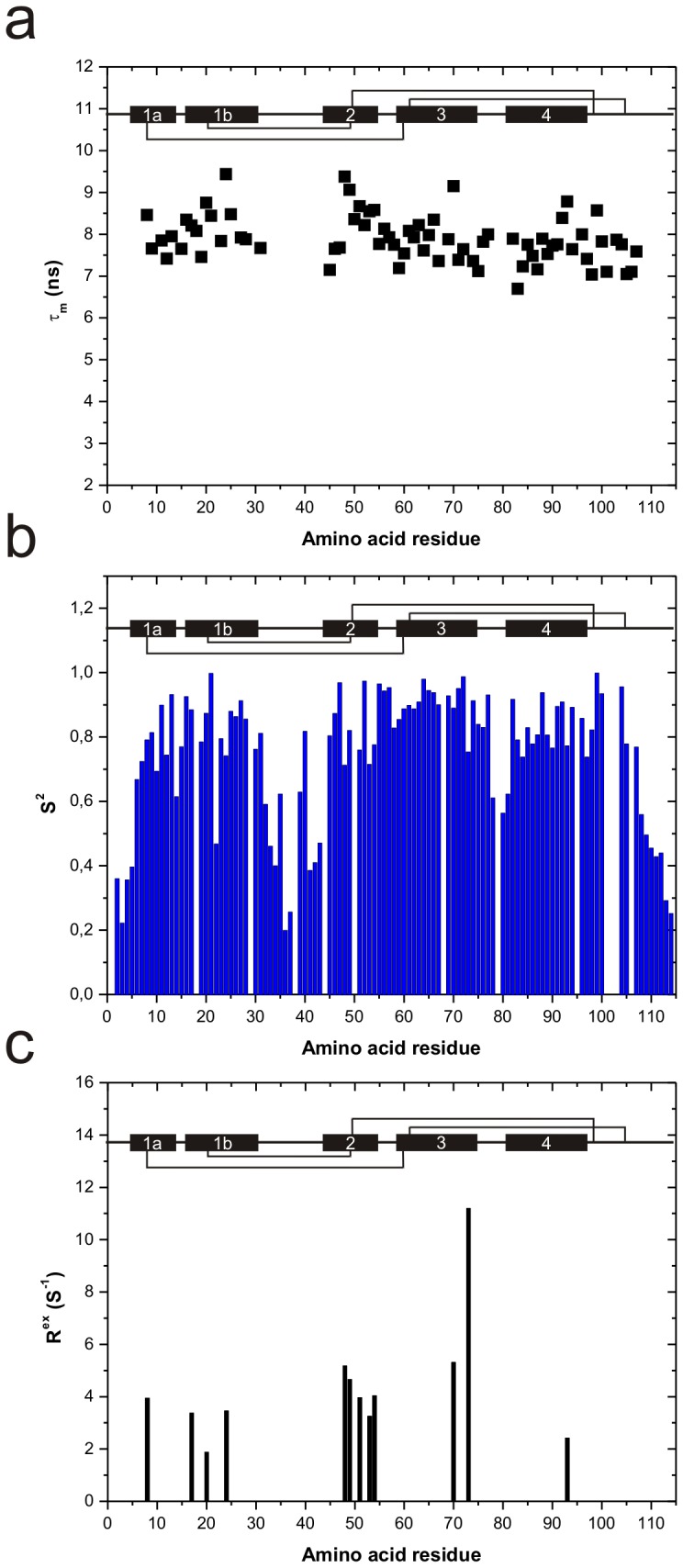
Ber e 1 backbone dynamics. a) Residue-specific overall tumbling time (τ_m_) values for 77 of 114 residues. The relatively uniform τ_m_ values indicate isotropic tumbling of Ber e 1. b) Order parameter, S^2^, of the N-H bond vector on a per residue basis. An S^2^ value of 1 equals a completely rigid N-H vector, whereas an S^2^ value of 0 implies complete rotational freedom of the N-H vector. Some flexibility is observed in the hypervariable loop, and the N- and C-terminal, as well as the non-native loop show high flexibility. c) R_ex_ parameter, showing the residues where µs-ms dynamics could be identified. Slow dynamics is largely located to the interface of helix 1b and 2, as well as at the end of helix 3, leading into the hypervariable loop. The secondary structure elements, as well as the cysteine linkage are indicated in the top of each figure.

R_1_, R_1ρ_ and ^15^N-{^1^H} NOE values of Ber e 1 could be extracted from all except 7 of the total 114 amino acids, these seven were either prolines or had severe overlap in the ^1^H-^15^N correlated spectra. The transverse relaxation rate (R_2_) was extracted according to equation 1 (Material and Methods). T_1_ (1/R_1_) and T_2_ (1/R_2_) relaxation times and ^15^N-{^1^H} NOE values are available in Supporting information Figure S1. The apparent overall rotational correlation time (τ_m_) was calculated from the square root of the T_1_/T_2_ ratio [38], [39]. For 77 residues apparent τ_m_, values could be calculated accurately (Figure 3a) and the remaining residues were discarded due to slow internal motions (see materials and methods) or by overlap in the NMR spectra. These residues were given an average calculated τ_m_, value in the further analysis.

The experimentally derived τ_m_ values of Ber e 1 are presented in Figure 3a. To validate the experimental conditions the theoretical τ_m_ value based on the structure of Ber e 1 was extracted through hydrodynamic calculations [40], yielding an isotropic overall rotational correlation time of 6.9 ns and an axial symmetry (Δ = 2D_zz_/(D_xx_+D_yy_) of 1.4. The experimental τ_m_ values with an average τ_m_ of 7.9 ns are thus slightly higher than expected. In addition the rather uniform τ_m_ values indicate a molecular tumbling which is close to isotropic, which also deviates from the hydrodynamic calculations. The experimentally derived τ_m_ and the apparent isotropic tumbling is likely caused by O-linked glycosylation that occurs upon expression in *Pichia pastoris* [12]. Strong NOEs in the ^1^H-^15^N NOESY spectrum from SER101 to a sugar moiety (data not shown), suggests glycosylation of this residue. In addition, SER 96 and SER110 also appear to be glycosylated, but to a smaller extent. From mass spectrometry data it was previously concluded that the glycosylation by *Pichia pastoris* is non-uniform with varied size distribution of attached sugar moieties [12].

Ber e 1 exists in many isoforms, and the isoform used in this study has been proven to also exist as a dimer [70]. Another explanation to the deviating experimental and theoretical τ_m_ values can therefore be that Ber e 1 exists as a mixed population between monomers and dimers, and the observed τ_m_ values reflect a population averaged tumbling time. If this is the case the relative small difference in theoretical and experimental τ_m_ values reflects a relative small population of Ber e 1 dimers.

There is no evidence of glycosylation in the wild type protein. However, the glycosylation of rBer e 1 is not likely to affect the overall structure as evident from the similar fold of rBer e1 compared with other 2S albumins. Additionally, the rBer e 1 and wt Ber e 1 share the same secondary structure, disulfide pattern and thermal stability as well as allergenic properties [12], [34].. With the exception of SER 101, which shows the strongest NOE to a sugar in addition to severe line broadening, the glycosylation of other residues in Ber e 1 seems to have negligible effect on the backbone dynamics.

Relaxation data were fitted to either S^2^ and τ_i_ or S^2^ and R_ex_ models given in the model-free software [41], [42]. The order parameter (S^2^)describes the degree of flexibility of the N-H bond vector on a ps-ns timescale and can have values between zero for a fully flexible vector and unity for a rigid vector, and is presented in Figure 3b, whereas R_ex_ describes a conformational exchange process that occurs on a µs-ms timescale (Figure 3c). SER101 was omitted from the spin relaxation analysis since the attached glycan on this residue affects the relaxation properties, and acts to increase µs-ms dynamics of the backbone for this residue.

The order parameters reveal that Ber e 1 is a fairly rigid molecule with S^2^ values around 0.8 for the structured part of the protein. The non-native loop is as flexible as the N- and C-terminals at the ps-ns timescale, with S^2^ values down to 0.2. Some degree of backbone flexibility is also observed in the hypervariable loop, as judged by the slightly decreased S^2^ values down to 0.6. Based on the S^2^ values the loop regions exhibit more dynamical processes than what is reflected in the structure ensemble of Ber e 1. This may reflect sampling of more flexible conformational states in the hypervariable region that are not captured in the calculated lowest energy structures, showing that measurement of the backbone dynamics is a good complement to NMR structure determination. In all known 2S albumin structures the backbone of the hypervariable loop is rather well defined despite its solvent exposure. Solvent exposed backbone segments are likely to have decreased S^2^ values, which is also the case in Ber e 1. Further investigations are required to establish if this holds true for all 2S albumins. Dynamics on the slow (µs-ms) time scale is mostly located to helices 1b and 2, as well as in the end of helix 3 leading into the hypervariable loop (Figure 3c). Altogether, the backbone dynamics measurements reveal that Ber e 1 samples structural states that are invisible in the NMR ensemble, and such states have been shown to be involved in ligand binding [43], [44]. This is particularly interesting for the hypervariable region as the dynamics could be a factor that enables IgE recognition, and therefore be involved in allergenicity.

No dynamical measurements on other 2 s albumins have been published, but the dynamics of the smaller homologous type 2 ns-LTP from wheat in complex with LPG has been investigated [45]. Like Ber e 1, the overall S^2^ values are around 0.8, however without decreased S^2^ values in the loop region corresponding to the hypervariable loop. The wheat LTP in complex with LPG does not show significant dynamics at the ms-µs timescale. This raises the interesting hypothesis that lipid binding may reduce protein dynamics.

### Electrostatic surface potential and Cu^2+^ interaction

The water soluble protein fraction from BN seeds, which contains Ber e 1 along with other proteins, has been reported to contain unusually high levels of divalent metals [17]. Interestingly, during the expression of large volumes of rBer e 1 in fermenters it was observed that the recombinant protein was able to bind supplementary metals with high affinity [10].

The stoichiometry between Ber e 1 and Cu^2+^ was determined using inductively coupled plasma mass spectrometry (ICP-MS). After incubation in a solution with 10-fold excess of Cu^2+^, followed by extensive dialysis almost equal concentrations of Cu^2+^ and protein were observed (Table 2). This result supports our NMR-based findings that the structure of Ber e 1 comprises a high-affinity Cu^2+^ binding site with a 1∶1 stoichiometry. For a Ber e 1 control not incubated in a Cu^2+^ solution stoichiometry between Ber e 1 and Cu^2+^ was determined to 65∶1, which shows that a small amount of trace metals from the *Pichia pastoris* fermentation remain bound to Ber e 1 even after extensive purification.

**Table 2:** ICP analysis of Cu^2+^ content.

	[Ber e 1] (µM) before dialysis	[Ber e 1] (µM) after dialysis	[CuCl_2_] (µM) before dialysis	[CuCl_2_] (µM) after dialysis	Ratio Ber e 1: Cu^2+^
Ber e 1+CuCl_2_	116	28.0	1160	21.0	4∶3
Ber e 1	116	-	-	1.78	65∶1

Copper ions (Cu^2+^) are paramagnetic, which can be utilized in NMR experiments where Cu^2+^ effectively bleaches out all NMR resonances within 8 Å proximity by paramagnetic relaxation enhancement [46]. Thus, residues within 8 Å from the copper binding site can be identified and subsequently used to estimate a Cu^2+^ binding site.

**Figure 4:**
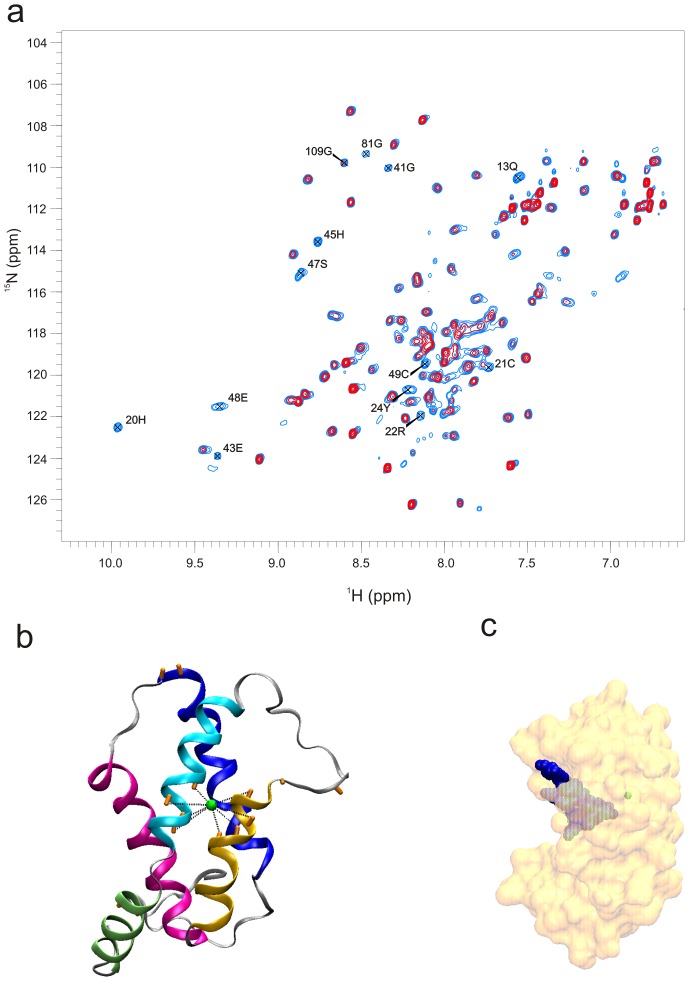
Ber e 1 copper interaction. a) Paramagnetic copper relaxation enhancement experiment on Ber e 1. The spectrum shown in black is recorded in the absence of copper, whereas the spectrum shown in red has copper added in a 1∶1 (Cu^2+^:Ber e 1) stoichiometry. N-H groups in the backbone affected by paramagnetic relaxation enhancement by the addition of Cu^2+^ in a 1∶1 ration are HIS 20, CYS 21, ARG 22, TYR 24, GLU 43, HIS 45, SER 47, GLU 48, CYS 49, and GLN 52. In addition N-H groups form sidechains (s.c) of GLN 11, 13, 28 and 83 are also affected by Cu^2+^ at this stoichiometric ratio. b) A model of the copper atom positioning in Ber e 1, based on the nearby residues identified in (a). The N-H backbone groups that are bleached are indicated in the structure as orange rods. c) Due to the slow dynamics around the copper binding site, the copper atom is engulfed into the core of the protein. Interestingly, its position is very close to the bottom of the hydrophobic cavity.

An NMR titration experiment exploiting the paramagnetic properties of Cu^2+^ was conducted, in which ^1^H-^15^N HSQC experiments were recorded in absence and presence of Cu^2+^ at different stoichiometric ratios (Figure 4a). At a 1∶1 stoichiometric ratio backbone crosspeaks from HIS 20, CYS 21, ARG 22, TYR 24, GLU 43, HIS 45, SER 47, GLU 48, and CYS 49 are completely bleached out by the paramagnetic enhancement effect of the Cu^2+^ ion. Due to the charged nature of Ber e 1 unspecific Cu^2+^ interaction will also occur, which will affect surface exposed atoms. Hence, at higher stoichiometric ratios more crosspeaks start to become affected, indicative of this weak non-specific binding. These measurements allowed for the mapping of an approximate copper binding site, where crosspeaks from residues that were completely absent at a 1∶1 stoichiometric ratio were used to model the Cu^2+^ binding site into the existing structure of Ber e 1 (Figure 4b). It is interesting to note that residues involved in µs-ms dynamics (Figure 3b), as well as residues involved in the copper binding site, are located in the interface between helix 1b and helix 2. In addition many of the residues close to the copper ion also form parts of the hydrophobic cavity (Figure 4c).

**Figure 5:**
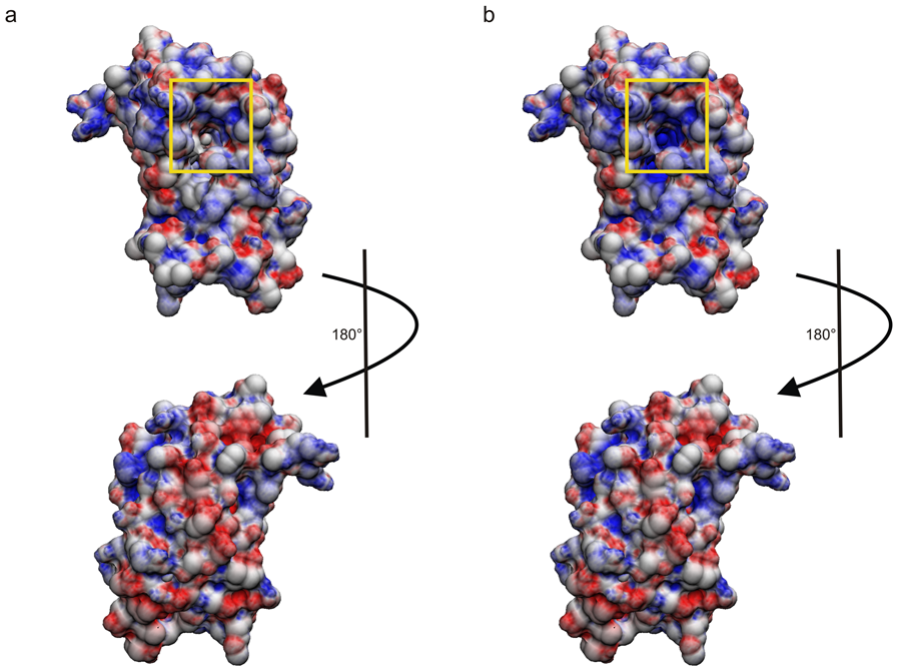
Electrostatic surface potential of the Ber e 1 in absence and presence of Cu^2+^. a) Electrostatic surface potential of the Ber e 1 at pH 7 in absence of Cu^2+^. b) Electrostatic surface potential of the Ber e 1 at pH 7 in presence of Cu^2+^. The entry of the hydrophobic cavity is highlighted with a yellow square. The protein shows an overall positive charge on the side of the protein that comprises the hypervariable loop and the entry to the hydrophobic cavity. In contrast, the other side of the protein, in particular helix 1a and most of helix 1b and 2, is negatively charged. The presence of histidines, taken together with the slow dynamics and the overall negative charge between helix 1b and 2, suggests that Cu^2+^ would enter the molecule from the negatively charged side of the protein. However, binding of Cu^2+^ only to a small degree changed the net surface charge on the helix 1b-2 side of the protein. The largest difference in surface potential is observed within the hydrophobic cavity; tuning the surface potential inside the cavity from neutral to more positive charge.

To further elucidate the impact of copper binding we calculated the electrostatic surface potential in the presence and absence of Cu^2+^ using APBS [47] (Figure 5a and b, respectively). At neutral pH the side of Ber e 1 consisting of helices 1a, 1b and 2 form a largely negatively charged surface, whereas helices 3 and 4 on the other side of the molecule to a large extent form a positively charged surface, making Ber e 1 a polarized molecule. No major differences in electrostatic potential are observed on the surface of the protein when Cu^2+^ is introduced, however, a significant change in potential is observed deep inside the hydrophobic cavity (Figure 5). When the copper is modeled into the structure based on the bleaching of HN-resonances that are near the copper atom in the paramagnetic experiment, the binding site appears to be buried inside the protein. It is therefore likely that the conformational fluctuations observed through the µs-ms dynamics in the nearby residues control Cu^2+^ access to the binding site, which is in the vicinity of the only two Histidines in Ber e 1. The copper binding site is also very close to the bottom of the hydrophobic cavity, but given the overall positive charge and the hydrophobic nature on the opposite side of the protein, it is unlikely that the Cu^2+^atom would travel through the hydrophobic cavity in order to bind to the Histidines. As an effect of the Cu^2+^ ion not being bound on the surface of the protein, the biggest difference in electrostatic surface potential is seen inside the hydrophobic cavity, which becomes more positively charged upon Cu^2+^ binding, whereas only minor changes in the electrostatic potential are observed on the surface of the protein (Figure 5). Thus, binding of copper or other divalent ions potentially influences Ber e 1 lipid binding, and hence its allergenic properties.

### Conclusions

The structure of Ber e 1 reveals a hydrophobic cavity suitable for lipid interaction, which potentially mediates its allergenic properties. The hydrophobic cavity is comparable to the lipid binding site of the homologous type 2 ns-LTP from wheat both in size and location. ^15^N-spin relaxation reveals that the backbone in the hypervariable region and helix 1b and 2 undergo dynamics on µs-ms timescale, which is indicative of regions suitable for interactions with other molecules. Ber e 1 is, to our knowledge, the first example of a 2S albumin proven to specifically interact with Cu^2+^ in a 1∶1 stoichiometry with high affinity. The location of the Cu^2+^ binding site is buried inside the protein, thus the Cu^2+^ ion does not significantly alter the electrostatic potential on the outer surface of the protein. However, the milieu of the hydrophobic cavity is affected by the binding of the Cu^2+^ ion. This suggests a linkage between electrostatic and hydrophobic binding activity of Ber e 1 that may alter its allergenic properties, given the fact that a BN lipid fraction is required for an allergenic response in mice.

## Materials and Methods

### Protein expression and purification

Uniformly labeled ^13^C/^15^N- and ^15^N-labbeled rBer e 1 were expressed as secreted proteins by *Pichia pastoris* [48] and purified as described earlier by Alcocer et al [12].

### NMR spectroscopy

The NMR samples were prepared by dissolving 7 mg of lyophilized Ber e 1 into 500 µl aqueous solution containing 20 mM potassium phosphate and 1 mM NaN_3_ to prevent bacterial growth. Two Ber e 1 samples, one ^13^C/^15^N-labelled, and one ^15^N-labelled, were dissolved with a H_2_O∶D_2_O ratio of 9∶1, and one ^13^C/^15^N-labelled sample was dissolved in phosphate buffer in D_2_O only. The pH for all samples was adjusted to 5.8 with HCl or NaOH.

NMR experiments were conducted on a Bruker DRX-600 spectrometer equipped with a triple resonance (^1^H/^13^C/^15^N) cryoprobe with Z-gradient capability and a Bruker AMX2-500 spectrometer equipped with a triple resonance (^1^H/^13^C/^15^N) probe with XYZ gradient capability. Before the start of each experiment the temperature was calibrated to 303 K using the chemical shift of a water/DSS sample. ^13^C and ^15^N chemical shifts were referenced indirectly using the gyromagnetic ratio of ^13^C/^1^H and ^15^N/^1^H [49]. The backbone and side chain resonances were assigned previously [9]. Assignment was further confirmed by 3D ^13^C- and ^15^N- edited NOESY experiments with 80 and 100 ms mixing time respectively, as well as a 3D ^13^C edited NOESY with 80 ms mixing time optimized for the methionine side chains. NOESY spectra were processed in NMRPipe [50] and analyzed using CCPN Analysis [51].

### ^15^N-spin relaxation

All ^15^N-spin relaxation experiments were conducted at a ^1^H frequency of 600 MHz (^15^N frequency of 60.8 MHz) at 303 K.

Longitudinal ^15^N-spin relaxation rates (R_1_) were measured via a standard pulse sequence [38], and transverse relaxation rates (R_2_) were extracted through R_1ρ_ experiments [38], [52] modified to incorporate a WATERGATE sequence [53] to improve water suppression. The recovery delay between transients was 2 seconds in the R_1_ and R_1ρ_ experiments. R_1_ relaxation experiments were recorded in an interleaved manner with (randomly distributed) relaxation delays of 10*,150, 300, 450, 600*, 700, 900 and 1200 ms (* duplicate time points). Relaxation delays of 10*, 50, 80, 100*, 120, 150, 170 and 190 ms were used in R_1ρ_ experiments with a spin lock field of 1560 Hz. The spin lock that is active during the R_1ρ_ relaxation delay may cause substantial heating of the sample. Systematic temperature differences between spectra with differing R_1ρ_ relaxation delays were avoided by applying a compensating ^15^N spin lock at the end of the recycle delay for a period of T′ = T_max_−T, where T_max_ is held constant at 190 ms and T is the R_1ρ_ relaxation delay.

The ^15^N-{^1^H} hetero-nuclear NOE experiment values were recorded with a standard pulse sequence [38] modified with a Watergate sequence [53] and a water flipback pulse to minimize the effect of the slowly relaxing water magnetization on the NOEs measured for amides with rapidly exchanging protons [54]. Two sets of experiments were recorded in an interleaved manner. Proton saturation was achieved by a train of 120° pulses separated by 5 ms during the 5 second long recovery delay. Spectra recorded without proton saturation utilized a 5 seconds recovery delay. The residue specific hetero-nuclear ^15^N-{^1^H} NOE values were determined from cross peak intensity ratios of spectra recorded with and without saturation of the ^1^H resonances. The experimental T1 and T2 relaxation times, together with the hetero-nuclear ^15^N-{^1^H} NOE values are presented in supporting information Figure S1.

### ^15^N relaxation data processing and analysis

Linear prediction was applied in the indirect dimensions. All dimensions were apodized by phase-shifted square sine bells and zero filled to final matrix size of 2048 by 256 points. Integration of cross peaks, curve fitting and extraction of relaxation rates were done with the rate analysis package within NMRViewC [55].

R_1_, R_1ρ_ and ^15^N-{^1^H} NOE data were integrated over an area corresponding to an ellipsoid with 8×4 Hz in the ^1^H and ^15^N dimensions, respectively. The use of small integration areas, essentially just the crest of the cross-peak, has the advantage that the noise is averaged, compared with pure intensity measurements. At the same time partially overlapping peaks can often still be reliably integrated provided that the small integration areas do not overlap [56], [57]. By using this method almost all cross-peaks could be utilized to extract the relaxation properties of Ber e 1 resonances.

The R_1_ and R_1ρ_ rates were determined by fitting the peak volumes to a two-parameter single exponential decay function and errors in relaxation rates were estimated through Monte Carlo simulations of the relaxation data. The residue specific R_2_ relaxation rates were extracted from the R_1ρ_ relaxation rates [58] according to the equation (1);
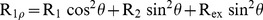
(1)Where R_1_ and R_2_ is the longitudinal and exchange-free transverse relaxation rate constants; R_ex_ is the conformational exchange contribution to transverse relaxation; θ = arctan(ω_1_/Δω) is the tilt angle between the reduced static magnetic field Δω = ω−ω_0_ and the effective field ω_e_ = (Δω^2^+ω_1_^2^)^½^ in the rotating frame; ω is the spin lock frequency; ω_0_ is the population average ^15^N Larmor frequency; and ω_1_ is the precession frequency around the spin lock field.

Experimental T_1_, T_2_ and ^15^N-{^1^H} NOE values of Ber e 1 were fitted to either S^2^ and τ_i_ or S^2^ and R_ex_ models given in the model-free software [41], [42]. The quality of the fits of experimental relaxation parameters to the different models was assessed from a χ2 comparison of calculated and experimental relaxation parameters and selection of the best model was then performed via statistical F-tests.

### Structure calculations

Structure calculations of the Ber e 1 molecule were performed in three parts. In part I, an initial (rough) structure of the molecule was created using the in-house written software Protein Constructor [59], [60] and torsion angles of the protein backbone only. Protein Constructor can generate a rough protein structure for a given sequence and required conformation (α-helix, β-strand, random coil) or from a list of specified consecutive backbone torsions angles. The backbone torsions angles of the protein were calculated based on the NMR chemical shifts for Ber e 1 published previously [9] BMRB accession bmrb6529. To be able to compare and evaluate the extracted backbone torsions angles we used both the program PREDITOR [61] and the program TALOS+ [62]. The initial structure was then used as starting structure in XPLOR-NIH v.2.29 [63] for further calculations.

In part II, Xplor-NIH was used to connect the 4 disulfide bridges in the initial structure implemented as covalent bonds between residue 8–60, 21–49, 50–98 and 62–105. A high temperature molecular dynamics simulation without any additional restraints was then performed in order to independently generate new starting structures. Three new structures with large rmsd were chosen as starting conformation for the subsequent simulated annealing protocol. For the simulated annealing calculations a set of experimental restraints was used: backbone torsion angles as mentioned above, NOE-distance restraints, ^3^J _HNHA_ coupling constants which were extracted using CCPN Analysis [51] and CA/CB chemical shifts. 100 structures were calculated from each of the three starting conformations and the 10 lowest energy, accepted structures from each calculation were subsequently submitted for further refinement. All molecular dynamics calculations in part II were performed using an IVM algorithm [64] in torsion angle space.

In part III, the 30 low energy structures from the previous simulated annealing were refined according to a gentle refinement protocol as described in [65]. The protocol consists essentially of a 30 ps constant temperature simulated annealing molecular dynamics calculations in cartesian space. The last 10 ps of each recorded trajectory were averaged and energy minimized to produce a final structure. Structures were then compared with respect to rmsd and the 12 structures with the lowest rmsd value were accepted as the final structure ensemble. Representatives from all three starting conformations are found in the final ensemble, which show a good structural convergence based on the experimental data. Final structures and intermediate results were visualized in VMD [66]. The methodology for structure calculations is described in detail in supporting information Protocol S1.

### Inductively coupled plasma mass spectrometry

Inductively coupled plasma mass spectrometry (ICP-MS) was used to determine the amount of Cu^2+^ bound to Ber e 1. Two samples with a concentration of 116 µM rBer e 1, one in Milli-Q water with a ten-fold excess of CuCl_2_, and a control sample with only Milli-Q water were incubated overnight. The copper sample was then extensively dialyzed against Milli-Q water using a Vivaspin 6 ultrafiltration unit with a MWCO of 3 kDa. After dialysis the protein concentration was determined using A_280_, and the Cu^2+^ concentration was determined using ICP-MS. Based on the protein and Cu^2+^ concentration the stoichiometry of binding could be determined.

### *In silico* structure analysis

SiteHound server [31] computes interactions between a chemical probe and a protein structure; therefore it is capable of predicting ligand binding sites in proteins. Both the methyl carbon and aromatic carbon probes within the software were used to identify hydrophobic binding sites. The significance of the predicted hydrophobic binding site was selected by two criteria, (i) the binding site should be present in all 12 structures, and (ii) the volume of the binding site should exceed 40 Å^3^.

Peptide Cutter of the ExPASy Server [67]was used to map the theoretical pepsin cleavage sites on the Ber e 1 primary structure. These sites were then visualized on the three dimensional structure of Ber e 1 using VMD [66]. To further probe the solvent accessibility of the cleavage sites, the surface accessibilities of the heavy atoms in the lowest energy structure of Ber e 1 were extracted using NACCESS [68] with a probe radius of 1.4 Å.

The DALI server [36] was used to compare the tertiary fold of Ber e 1 with other structures in the Protein Data Bank (PDB). The lowest energy structure of Ber e 1 was chosen as a representative in the comparison of the atomic coordinates in the DALI search.

The DIFFC program within the DASHA software [40] was used to characterize hydrodynamic properties and to calculate nuclear spin relaxation parameters for molecules with known structures. DIFFC uses a bead model approximation, which assumes that the molecule is a rigid body, represented by a number of frictional points with particular radii, the so called beads. The hydrodynamic properties of a molecule estimated in this way are highly dependent on the size chosen for the beads. A bead size of 3.4 Å for all heavy atoms was chosen, as described in [69] and the hydrodynamic calculations were performed at 303 K using the lowest energy structure of Ber e 1.

The modeling of Ber e 1:Cu^2+^ complex was performed using X-PLOR. H^N^ atoms of the backbone that were significantly affected by paramagnetic relaxation enhancement (Figure 5) were used as distance constraints (1.8–8 Å) to the Cu^2+^ ion. Before the start of energy minimization the Cu^2+^ ion was placed in an approximate location based on the NMR data. Subsequently, a short energy minimization, as described in part III in the structure calculation, was performed allowing the Cu^2+^ ion to be positioned so that all constraints were fulfilled.

## Supporting Information

Protocol S1 **Detailed description of structure determination protocol.**
(RTF)

Figure S1 **Figure of T1 and T2 relaxation times and ^15^N-{^1^H} NOE values.**
(TIF)
