# Supplementary material for: Solution Structure, Copper Binding and Backbone Dynamics of Recombinant Ber e 1–The Major Allergen from Brazil Nut
Source: PLoS One. 2012 Oct 4;7(10):e46435. doi: 10.1371/journal.pone.0046435 (PMC3464261; doi:10.1371/journal.pone.0046435)
Supplement: Protocol S1 — Detailed description of structure determination protocol. (RTF) [file pone.0046435.s001.rtf]

Supplementary information


Structure calculation
Part I.
Structure calculations of Ber e 1 were performed in three parts. Below follows a detailed description of each part in the structure determination.

Part I
ö and ø backbone torsion angles restraints were derived from the 1H, 13C and 15N chemical shift values (BMRB accession number 6529) using both the PREDITOR software [1] and the TALOS+ software [2]. For residues with chemical shifts and output from PREDITOR/TALOS characteristic of an á-helix the ö and ø backbone torsion angles restraints were kept at values predicted by the programs, but with error margins increased to ± 30. Residues where the predicted ö and ø values were close to but not evidently indicating helical conformation the error margin was increased to ± 45. Those regions were primarily located on the junctions between sequences with strict helical conformations. For other regions, with evidently random coil indication the error margin was kept as given by the programs.
Based on the output of PREDITOR and TALOS+ the Ber e 1 structure was divided into a few structural elements consisting of either random coil (RC) or á-helix (H). The structural elements identified were; residues 1-4 (RC), residues 5-15 (H1a), residues 16-17 (RC), residues, 18-30 (H1b), residues 31-44 (RC), residues 45-56 (H2), residues 57-58 (RC), residues 59-75 (H3), residues 76-81 (RC), residues 82-97 (H4) and residues 98-114 (RC). 
An initial (rough) structure of Ber e 1 was then created using our in-house software Protein Constructor and torsion angles of the protein backbone. Protein Constructor has previously successfully been used for different kind of treatment of protein structures[3,4]. The structure was then energy minimized in XPLOR-NIH using 2000 steps of the Powell minimization algorithm.

Part II
XPLOR-NIH was used to create the 4 disulfide bonds in Ber e 1 as covalent bonds between residues 8-60, 21-49, 50-98 and 62-105. This modified topology was then used in all subsequent structure calculations. To have some independent mechanism for proving a possible convergence of the calculated final structures a high temperature molecular dynamics (without any constraints) was performed in order to create three new starting structures with a large rmsd between the different structures. The three new structures were then used as starting conformations in the subsequent simulated annealing (SA) protocol.
For the simulated annealing molecular dynamics calculations using XPLOR-NIH all available restraints provided by NMR experiments were applied: 1068 NOE-distance restraints, 88 3J HNHA coupling constants, 112 CA/CB chemical shifts and the calculated backbone torsion angles ö and ø. The NOE constraints comprised 634 distances between 1.7 and 2.8 Å, 321 between 2.8 and 3.4 Å and 113 greater than 3.4 Å. The calculation of the distances were performed within CcpNmr Analysis [5] based on the experimental 3D 15N- edited NOESY-HSQC and 3D 13C- edited NOESY spectra. The conversion of peak volume into distance restraints were according to default settings. Of the total amount of 1068 distance restraints 606 are intraresidual whereas the remaining 462 distances were interresidual. 
More generous lower and upper bounds of the distance restraints than suggested by CcpNmr Analysis were applied. The lower bounds were kept uniformly to the contact value of 1.8 Å whereas the upper bounds were calculated for every distance according to Folmer et al.[6], so that MD calculations could be smoothly performed. Additionally, 88 3JHNHA-coupling constants obtained from NMR measurements were used directly in the XPLOR-NIH force field for constraining ö-torsion angles of the backbone and 112 restraints for the CA/CB chemical shift potential. Before performing simulated annealing calculations the 4 disulfide bridges were built into the initial structure as described above. The SA calculations were carried out using the IVM implementation in torsion-angle space. From each of the three randomized starting conformations 100 structures of Ber e 1 were calculated using SA protocol going from a high temperature of 3000K to the final temperature of 20K in decremental steps of 20K. The high temperature period of the SA protocol consisted of 20000 steps followed by 400 steps at each of the cycles at lower temperature. The resulting 100 structures were energy minimized using 2000 steps of Powell minimization. 10 of the accepted lowest energy structures originating from each starting conformer were then submitted for further refinement.
 Part III
Refinement of the 30 accepted structures obtained from Part II was performed within the program XPLOR-NIH using the “gentle refinement” protocol. The procedure was carried out by running 30 ps molecular dynamics calculations at 300K in cartesian coordinate space and recording the trajectory. After the initial equilibration average coordinates from the last 10ps of the molecular dynamics calculation were computed and refined using 2000 Powell energy minimization steps. The resulting structures from different starting conformations were compared and ordered by means of pairwise rmsd caclulations. 12 structures with the smallest pairwise rmsd values were chosen as the final structure ensemble. During the refinement the forcefield and the restraining terms were identical to the high temperature simulated annealing (Part II), with the exception that calculations were performed in cartesian coordinates space.

Supplementary Figure S1
a) T1 and T2 relaxation times. T1 relaxation times are shown in red triangles and T2 relaxation times are shown as black triangles.
b) 15N-{1H} heternuclear NOE.

1. Berjanskii MV, Neal S, Wishart DS (2006) PREDITOR: a web server for predicting protein torsion angle restraints. Nucleic Acids Research 34: W63-W69.
2. Shen Y, Delaglio F, Cornilescu G, Bax A (2009) TALOS plus : a hybrid method for predicting protein backbone torsion angles from NMR chemical shifts. Journal of Biomolecular Nmr 44: 213-223.
3. Zdunek J, Martinez GV, Schleucher J, Lycksell PO, Yin Y, et al. (2003) Global structure and dynamics of human apolipoprotein CII in complex with micelles: Evidence for increased mobility of the helix involved in the activation of lipoprotein lipase. Biochemistry 42: 1872-1889.
4. Gangabadage CS, Zdunek J, Tessari M, Nilsson S, Olivecrona G, et al. (2008) Structure and dynamics of human apolipoprotein CIII. Journal of Biological Chemistry 283: 17416-17427.
5. Vranken WF, Boucher W, Stevens TJ, Fogh RH, Pajon A, et al. (2005) The CCPN data model for NMR spectroscopy: development of a software pipeline. Proteins 59: 687-696.
6. Folmer RHA, Nilges M, Papavoine CHM, Harmsen BJM, Konings RNH, et al. (1997) Refined structure, DNA binding studies, and dynamics of the bacteriophage Pf3 encoded single-stranded DNA binding protein. Biochemistry 36: 9120-9135.
