# Supplementary figures and images for: Solution Structure, Copper Binding and Backbone Dynamics of Recombinant Ber e 1–The Major Allergen from Brazil Nut
Source: PLoS One. 2012 Oct 4;7(10):e46435. doi: 10.1371/journal.pone.0046435 (PMC3464261; doi:10.1371/journal.pone.0046435)

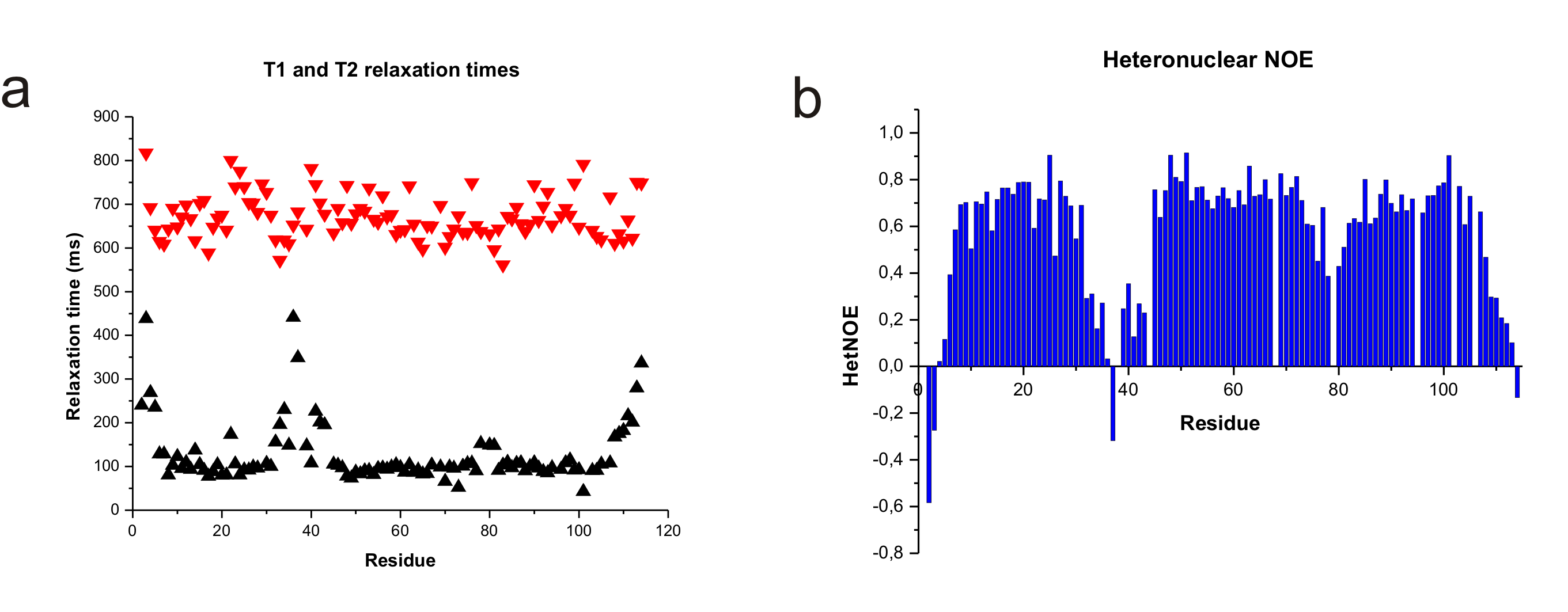

Supplement: Figure S1 — Figure of T1 and T2 relaxation times and 15N-{1H} NOE values. (TIF) [file pone.0046435.s002.tif]
